# The Herb-Drug Interaction of Clopidogrel and Xuesaitong Dispersible Tablet by Modulation of the Pharmacodynamics and Liver Carboxylesterase 1A Metabolism

**DOI:** 10.1155/2018/5651989

**Published:** 2018-10-18

**Authors:** Shitang Ma, Guoliang Dai, Xiaolin Bi, Meirong Gong, Chenggui Miao, Hao Chen, Liangliang Gao, Weiman Zhao, Tianjun Liu, Ningru Zhang

**Affiliations:** ^1^Life and Health College, Anhui Science and Technology University, China; ^2^Institute of Bio-Medical Engineering, Chinese Academy of Medical Sciences, China; ^3^Nanjing University of Chinese Medicine, China; ^4^The First Affiliated Hospital of Bengbu Medical College, China

## Abstract

**Objective:**

Clopidogrel and Xuesaitong dispersible tablet (XST) have been clinically proven to be effective for treating cardiocerebrovascular disease. The present study was to investigate the herb-drug interaction of Clopidogrel and XST by modulation of the pharmacodynamics and liver Carboxylesterase 1A(CES1A) metabolism.

**Methods:**

30 male SD rats were randomly divided into a control group (equal volumes of saline, 6 rats for mRNA analysis), a clopidogrel group (clopidogrel with dose 30 mg/kg), and a combination group (clopidogrel and XST, with dose 30 and 50 mg/kg respectively, each group continuous administration once daily for 30 days). The clopidogrel and combination group comprised 12 rats, with 6 designated for mRNA analysis and 6 for the pharmacokinetic study. The 2-bromo-3'-methoxyacetophenone- (MPB-) derivatized clopidogrel active thiol metabolite (CAMD) was measured by UHPLC-MS/MS for pharmacokinetics (n=6). The expression of CES1A mRNA was examined with real-time RT-PCR (n=6). Molecular simulation was used to investigate the inhibition effect of XST on the CES1A protein. The CAMD pharmacodynamics and CES1A metabolism were investigated to evaluated the herb-drug interaction.

**Results:**

Clopidogrel and XST coadministration appreciably increased the Cmax, AUC, and MRT of CAMD. However, the expression of CES1A mRNA was decreased accordingly. It also indicated that the bioactive components in XST had good interaction with the CES1A metabolism target by molecular simulation. The animal study indicated that clopidogrel and XST coadministration produced significant herb-drug interactions at active CAMD pharmacokinetic and CES1A metabolic enzyme aspect.

**Conclusion:**

30-days dose of coadministration altered hepatic CES1A protein and resulted in reduced plasma levels of active CAMD. both the decreased CES1A mRNA expression and the inhibition on the protein were due to the combination of XST, which accordingly upregulated the pharmacokinetics of plasma active CAMD.

## 1. Introduction

Clopidogrel, a second generation thienopyridine P2Y12 inhibitor, has been the standard-of-care for percutaneous coronary intervention (PCI) and/or acute coronary syndrome (ACS) [1–3]. Although the drug is generally considered safe and effective, many clinical studies have shown that approximately 5–40% of patients displayed inadequate antiplatelet responses [2, 4]. This result has increased platelet reactivity and cardiovascular events during treatment [5]. These patients need to seek alternative antiplatelet therapies [3, 6, 7], e.g., Chinese medicine combinations.

**Figure 1:**
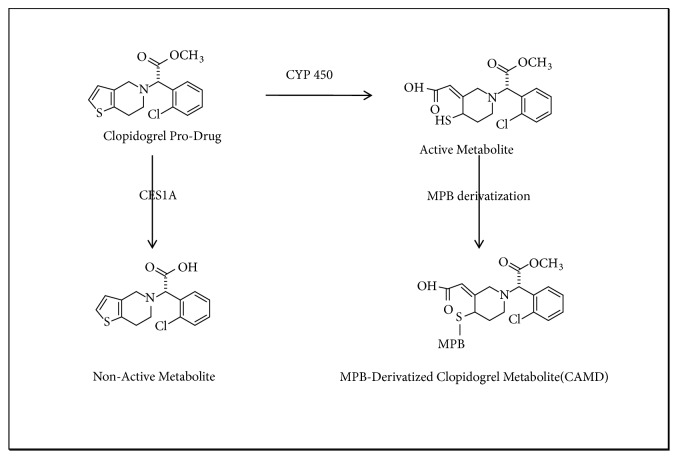
Formation of the active CAMD metabolite.

Clopidogrel is an inactive prodrug that requires enzymatic conversion by a number of carboxylesterases (CESs) enzymes and cytochrome P450 (CYP) enzymes [8–10]. During clopidogrel metabolism [11, 12], carboxylesterase 1A (CES1A) begins by hydrolyzing approximately 85-90% of the prodrug to an inactive carboxylic acid metabolite [13]. A portion of the prodrug is transformed into the inactive 2-oxo-clopidogrel [6], and the remainder is oxidized to the active thiol metabolite by CYP enzymes (Figure 1) [14, 15]. Only approximately 2% of the clopidogrel dose reaches systemic circulation where it becomes available through irreversible binding to the platelet P2Y12 receptor to have an antiplatelet aggregation effect [16].

Our previous study found that multiple dose (30 days) of clopidogrel altered hepatic CES1A in rats, which resulted in elevating the serum inactive carboxylic metabolite [17]. When a Chinese medicine recipe containing* Salvia Miltiorrhiza, Radix Notoginseng, and Borneol *combined with FDDP, the activity of CES1A was partially inhibited based on molecular simulation experiments [18], which have the pharmacokinetic result of decreasing levels of the inactive carboxylic acid metabolite [19].

Xuesaitong (XST, Chinese drug Z20050467), extracted from* Panax notoginseng (Burk.) F.H. Chen* (Sanqi), was widely used in TCM hospitals [20, 21]. Ginsenoside Rg1, Rd, and notoginsenoside R1 were its main active components [22, 23]. XST was used to treat cerebral infarction and ischemia, coronary heart disease, and atherosclerosis [24, 25]. However, there have not been any publications describing a herbal-drug effect of clopidogrel with XST through modulation of target metabolism and pharmacokinetics. The present study investigated the rationale of combined applications and the drug-herb effects on target metabolism (by RT-PCR for the CES1A mRNA expression and molecular simulation for the metabolic enzyme) and pharmacokinetics of CAMD(by UHPLC-MS/MS).

## 2. Experimental

### 2.1. Materials and Methods

#### 2.1.1. Reagents and Materials

The 2-bromo-3'-methoxyacetophenone- (MPB-) derivatized clopidogrel thiol metabolite (CAMD, lot No. 5-MNZ-195-23), MPB (lot No. 151910100), and internal standard (IS, guanosine, lot No. 111977-201501) were purchased from the Toronto Research Chemicals, Sigma, and the Chinese National Institute for the Control of Pharmaceutical and Biological Products (NICPBP), respectively. XST tablets (0.5 g/tablet, lot No. DHB1606, expiring before May 2018), containing 13.6 mg/g ginsenoside Rg1, 8.6 mg/g ginsenoside Rd, and 7.8 mg/g ginsenoside R1, were manufactured by the Yunnan Dali Medicine Factory (Yunnan, China). Clopidogrel tablets (25 mg/tablet, lot No. AA20150207, expiring before Jan 2018) were manufactured by Shenzhen Salubris Pharmaceuticals Co. (Shenzhen, China). Acetonitrile and methanol were HPLC grade (Merck, USA). The ultrapure water used for UHPLC-MS/MS was from a Milli-Q water purification system (Millipore, USA).

The RNAiso Plus reagent, PrimeScript™ II 1st Strand cDNA Synthesis Kit, SYBR Premix Ex Taq™ II kit and primer were provided by the TaKaRa Biotechnology Company (Takara, Japan).

### 2.2. Animal Treatment

Thirty male SD rats, body weights 220-300 g (License No. SCXK, Jiangsu Province, China, 2014-0007) were purchased from Suzhou Industrial Park Aier Matt Technology Co. Ltd. (Suzhou, China). All rats were pathogen-free and acclimated for at least one week. The rats were housed in an environmentally controlled room with a temperature of 20±2°C, light from 06:30 h to 18:30 h, and humidity of 60 ±5%. All rats were fed standard rodent chow and water ad libitum. This procedure was approved by the Animal Ethics Committee of the Nanjing University of Chinese Medicine.

Three groups were randomly assigned according to a parallel study. The control group consisted of 6 rats (oral administration of equal volumes of saline) for mRNA analysis. The clopidogrel group received 30 mg/kg of clopidogrel orally that comprised 12 rats, with 6 designated for mRNA analysis and 6 for the pharmacokinetic study. The combination group had 12 rats, with 6 allocated for mRNA analysis and 6 for the pharmacokinetics, and all orally received both clopidogrel at 30 mg/kg and XST at 50 mg/kg. The rats in each group were treated for 30 days. The rats fasted for 12 h before the experiment but had unlimited access to water. The drugs were suspended in saline before oral administration to rat.

### 2.3. Experimental Procedure

All rats were continuously intragastrically fed each drug for 1 month, as described in “animal treatment”. For the pharmacokinetic analysis, 6 rats each were selected from the clopidogrel and combination group. Blood plasma samples of approximately 150 *μ*L were collected in 500 mM of MPB pretreated EDTA centrifuge tubes from the fossa orbitalis vein at 0, 0.083, 0.25, 0.5, 0.75, 1, 2, 3, 4, 6, 8, 12, and 24 h before and after drug administration on the thirty-first day. Then, the samples were centrifuged for 10 min at 4 000 rpm, and the supernatant was transferred to labeled plastic vials and stored at −20°C until analyzed.

The remaining 18 rats from the three groups were used for mRNA analysis. After overnight fasting, the rats were sacrificed under anesthesia by i.p. administration of a 0.4 mL/100 g dose of a 10% chloral hydrate solution. The samples were finally collected as follows. The rat livers were removed and weighed. Portions of the liver samples were stored at −80°C for further mRNA biochemical assays [26].

### 2.4. The Stock Solutions and Plasma Sample Preparation

Master stock solutions were prepared by individually dissolving CAMD and IS in methanol at free-base equivalent concentrations of 1 000 *μ*g/mL. Working solutions were prepared from the stock solutions by dilution in methanol. All working solutions were stored at 4°C. For each run, calibration standards in drug-free rat EDTA plasma were freshly prepared in duplicate at concentrations of 66, 41.96, 10.24, 5.12, 2.56, 1.28, and 0.64 ng/mL for CAMD. QC samples were prepared at 49.5, 30, 1.8 ng/mL. All the standard calibration samples and QC samples were stored at -20°C.

All frozen standards and samples were thawed on wet ice before homogenization. A 50 *μ*L aliquot of each plasma sample and 10 *μ*L IS solution (860.00 ng/mL) were transferred into a 1.5 mL centrifuge tube where protein precipitation was performed by adding 200 *μ*L of methanol. The mixture was vortexed for 5 min and then centrifuged at 12 000 rpm for 10 min before the supernatant was transferred to the UHPLC-MS/MS for analysis [27, 28].

### 2.5. UHPLC-MS/MS Instrumentation and Conditions

Chromatographic separations were performed with an Agilent HPLC 1290 system (Agilent, USA) consisting of a quaternary pump, an online degasser, and an autosampler. The chromatographic separation was performed on a Phenomenex Gemini C18 reversed phase analytical column (110 Å, 3 *μ*m particle size, 2.0 mm I.D. × 100 mm). The mobile phase consisted of methanol and an aqueous solution of 0.1% formic acid (80:20;* v*/*v*) at a flow rate of 0.2 mL/min. The autosampler temperature was maintained at 4°C, and the injection volume was 5 *μ*L. The total LC run time was 5 min, with a column temperature of 30°C.

Detection of the analytes and IS was performed on a G6430 tandem quadrupole mass spectrometer (Agilent, USA) with an electrospray ionization (ESI) interface in positive ion mode. Multiple reaction monitoring (MRM) was used to monitor precursor to product ion transitions of* m/z* 504.1→211.7 for CAMD and* m/z* 284.1→152.1 for IS. the source parameters were a capillary voltage of 3.5 kV, a gas temperature of 350°C, and a gas flow of 10 L/min. The compound dependent parameters of fragmentor and collision energy were optimized at 130 V and 10 V for CAMD and at 90 V and 10 V for the IS, respectively. Dwell time set was 200 ms for CAMD and the IS. The resultant data were processed using MassHunter software (version B.05.00, Agilent).

### 2.6. UHPLC-MS/MS Method Validation

The method was validated in terms of linearity, accuracy and precision, selectivity, matrix effect (ME), recovery, and stability according to the guidelines for bioanalytical method development recommended by the US Food and Drug Administration and related literature for CAMD detection [14, 29].

### 2.7. Pharmacokinetic Analysis

The blood samples in the pharmacokinetic analysis were prepared, and the CAMD concentrations were assessed by the validated LC-MS/MS method. Pharmacokinetic parameters were calculated using the Drug and Statistic (DAS) 3.0 pharmacokinetic software (Chinese Pharmacological Association, Anhui, China).

### 2.8. RNA Extraction and Real-Time RT-PCR

Total RNA was extracted from 100 mg of the livers using the RNAiso Plus reagent (Takara, Japan) [13, 30]. Then, 0.5 *μ*g of the extracted RNA was reverse transcribed into first-strain complementary DNA (cDNA) using a PrimeScript™ II 1st Strand cDNA Synthesis Kit (Takara, Japan). Real-time RT-PCR was then performed using SYBR Premix Ex Taq™ II kit (Takara, Japan) on the Mx3000pTM Real-time RT-PCR system (Stratagene, Mx3000p, USA) following the 2^-ΔΔCt^ method. The following primers were used for the analysis of the rat samples: CES1A:5'-CTACCCACCTATGTGCTCCC-3'(sense) and 5'-GCCCAGGCGATACTGAATGAC-3'(antisense); *β*-ACTIN:5'- CACTATCGGCAATGAGCG -3'(sense) and 5'- AGGAGCCAGGGCAGTAATC -3'(antisense). The relative expression of the genes was normalized using Β-ACTIN as the internal reference.

### 2.9. Statistical Analysis

Each value obtained from experiments was expressed as the mean ± SE, n = 6. The mean comparisons for each group from the pharmacokinetic and mRNA Expression were performed using Student's* t*-test and one-way ANOVA respectively. Differences with* P* < 0.05 were considered statistically significant.

### 2.10. Molecular Simulation

Schrodinger Maestro 8.5 was used to investigate the molecular simulation. The XST bioactive components (ginsenoside Rg1, Rd and notoginsenoside R1) and the CES1A protein (PDB ID 1MX1) were prepared with LigPrep and protein preparation wizard, respectively. Then, the above materials were subjected to Glide based three-tiered in silico target screening strategy by two stages of the docking protocol, High Throughput Virtual Screening (HTVS), and Standard Precision (SP).

## 3. Results and Discussion

### 3.1. Pharmacokinetic Method Validation

**Figure 2:**
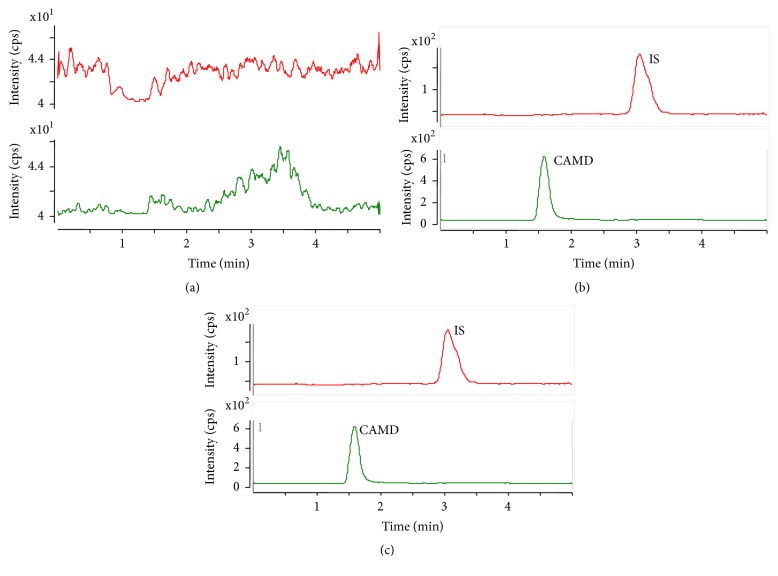
Chromatograms of CAMD and IS. (a) A blank rat plasma sample; (b) a blank plasma spiked with CAMD and IS at the LLOQ; (c) a rat plasma sample after an oral administration of XST (50 mg/kg) and clopidogrel (30 mg/kg) at intervals of 0.5 h.

The protein precipitation sample preparation in combination with UHPLC–MS/MS detection provided good selectivity for the CAMD analytes and the IS.  Figure 2 shows the typical chromatograms of a blank plasma sample, a blank plasma sample spiked with CAMD analyte at the LLOQ and the IS, and the TIC for the combination group plasma after multiple dose for 30 days at 0.5 h intervals. No significant interfering peaks were observed at the retention times of the analyte or the IS. The retention times for CAMD and the IS were 1.59 and 3.08 min, respectively.

The assay was validated over the nominal concentration range of 0.64-66.00 ng/mL. The calibration curve correlation coefficients (*R*^2^) were 0.998 8. A typical calibration curve equation was* Y* = 28.067* X* + 0.0018, which indicated a good fit of the calibration data to the regression lines. The lowest concentration at S/N ratios of 10 with the RSD <20% was taken as LLOQ and was found to be 0.64 ng/mL for CAMD (Table 1).

The extraction recoveries of the analytes from plasma at the three QC concentration levels were 90.20%-93.10%. The matrix effects at three QC levels were in the range of 92.40%-101.20% with RSD values below 6.70%. In this assay, the intra- and interday precisions were measured to be below 7.10% and 6.30%, respectively, with relative errors from −2.30% to 5.30% (Table 2).

The analytes were stable in the plasma samples for at least 6 h at room temperature or on ice. No significant degradation was observed when extracted plasma samples were kept at 4°C in the autosampler for up to 24 h.

### 3.2. Application of the Validated Assay to the Pharmacokinetic Study

**Figure 3:**
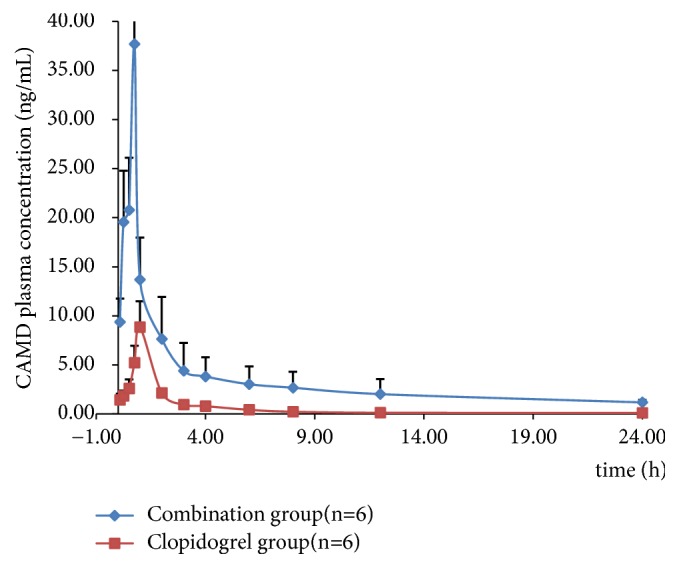
Mean plasma concentration–time profiles of CAMD in rats (n = 6) after continuous oral administration of clopidogrel (30 mg/kg) with or without XST (50 mg/kg).

The validated UHPLC–MS/MS assay was successfully applied to the quantitation of CAMD in rat plasma samples. The mean plasma concentration-time profiles are illustrated in Figure 3 and pharmacokinetic parameters are presented in Table 3.

The AUC_(0-24)_ and AUC_(0-*∞*)_ in rats after combinational administration of clopidogrel and XST were 84.13±4.72 and 107.03±5.31, which were significantly higher than that in clopidogrel group (15.91±2.93 and 16.31±3.15,* P*<0.05). The mean peak concentration (C_max_) was achieved with significantly high value of 37.71±6.34 in combinational group, which was detectable in normal rat plasma with low value of 8.92±2.63 in clopidogrel group (*P*<0.05). Similarly, the MRT of the CAMD were also significantly increased (*P*<0.05). There were no significant changes observed in T_max_, T_1/2_, and Vd/F of analyte concentration in the two groups.

In the present investigation, it was found that the pharmacokinetic parameters of CAMD in combinational group were different from those in clopidogrel rats. Clopidogrel and XST coadministration appreciably increased the Cmax, AUC, and MRT of CAMD (the active thiol metabolite). The above results indicated that combination with XST could increase the plasma concentrations of CAMD in rats.

### 3.3. CES1A mRNA Levels in Liver Tissues of Rats

**Figure 4:**
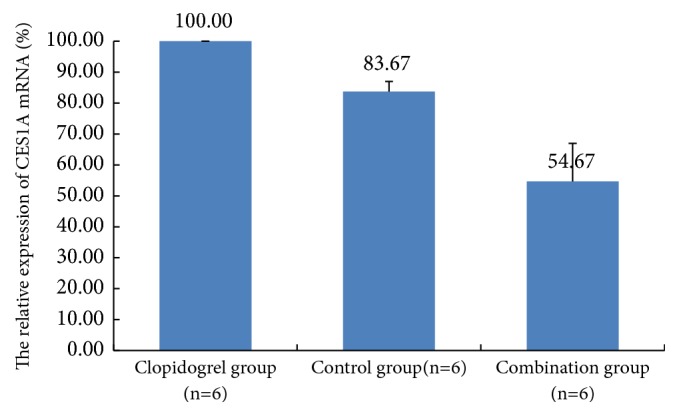
The relative expression of CES1A mRNA in rat livers after 30 days of administration in each group, as determined by RT-PCR.

The expression of the CES1A enzyme mRNA was measured by Real-Time RT-PCR in rat liver to evaluate the impact of the XST on clopidogrel hydrolysis. As shown in Figure 4, compared to the control group, there was a significant acute increase in the relative expression of CES1A mRNA in the clopidogrel group (83.67±3.30 versus 100), which indicated that 30-day dose of clopidogrel would increase the level of the CES1A mRNA in vivo [17]. This would upregulate the serum concentration of inactive clopidogrel carboxylic metabolites and downregulate the serum concentration of active CAMD metabolites correspondingly. Compared to the clopidogrel group, the mRNA expression level was downregulated when combined with XST (54.67±12.29 versus 100,* P*<0.05). As the CES1A enzyme is responsible for clopidogrel hydrolysis, changes in expression of the CES1A mRNA would result in altered plasma concentrations of the clopidogrel metabolite. As a result, the serum concentration of CAMD metabolites increased when combined with XST.

### 3.4. The Molecular Simulation between the XST and CES1A

The docking score of ginsenoside Rg1, Rd, and notoginsenoside R1 against CES1A protein from the molecular simulation were, respectively, -6.54, -7.12, and -8.13. Once the ligand-receptor complex formed, it adapted to the most stable conformation. The active site of the CES1A protein revealed that several molecular interactions could be considered responsible for the observed affinity. Hydrogen bonds could be found between the protein residue GLU815, LYS866, LUE838, and the XST ligand. These results suggest that the activity of the CES1A metabolic enzyme activity may be partially inhibited by XST. The molecular simulation results were consistent with the previous CES1A mRNA expression results.

Above all, great changes have took place in both pharmacokinetic parameters and CES1A metabolic enzyme aspect (by mRNA expression and molecular protein inhibition). Possible reasons for XST-clopidogrel interaction are complex and diverse, including gastrointestinal lesions that cause changes in drug absorption, changes in transporters responsible for uptake, efflux, and elimination, and changes in the metabolic enzymes which alter the clopidogrel metabolic rate.

Clopidogrel is an inactive prodrug that requires enzymatic conversion by carboxylesterases (CESs) and cytochrome P450 (CYP) enzymes. The liver is the organ responsible for drug metabolism enzymes. Patients have reduced hepatic metabolism of clopidogrel, via the CESs and P450 enzyme group. Currently, most of herbal medicines are administered via the oral route. While previous data showed that multiple dose of clopidogrel induced accumulation of the inactive clopidogrel carboxylic acid metabolite, the accumulation phenomenon was reduced by combination with the Chinese medicine FDDP [17]. Owing to decreased CES1A activity, the elimination of the parent drug can be changed when combined with herbal medicines.

Drug transporters also have a critical role in controlling drug exposure. Transporters are proteins facilitating the passage of drugs across biological barriers encountered during drug metabolism, among which P-glycoprotein (P-gp) can expel various drugs, resulting in multidrug resistance, and is likely to play a critical role in the uptake and absorption of substrate drugs. The intestinal absorption of ginsenoside Rg1, Rd, and notoginsenoside R1 (the main component of XST), is enhanced by the inhibition of P-gp activity. All of the above may contribute to the changes in pharmacokinetic behavior of CAMD in rats compared with when combination with XST. However, the proposed hypotheses still need further investigation and validation.

## 4. Conclusion

Sensitive UHPLC-MS/MS and RT-PCR technique were successfully used to characterize the clopidogrel and XST herb-drug interaction in rats. Clopidogrel and XST coadministration appreciably increased the Cmax, AUC, and MRT of CAMD (the active thiol metabolite) and decreased the CES1A mRNA expression. Animal studies indicated that clopidogrel and XST coadministration produced significant herb-drug interactions in pharmacokinetic and metabolic enzyme aspect. In a word, 30-day dose of coadministration altered hepatic CES1A, and this resulted in elevated serum CAMD levels. Decreased CES1A mRNA expression and elevated serum CAMD levels were due to the XST combination.

## Figures and Tables

**Table 1:** Mean matrix effect and recovery of CAMD in rat plasma (*n* = 6).

Concentration (ng/mL)	Matrix effect (%)	RSD (%)	Recovery (%)	RSD (%)
1.80	92.40	4.90	90.20	5.10
30.00	101.20	6.70	93.10	6.30
49.50	98.30	5.30	92.30	2.30

**Table 2:** Precision and accuracy of CAMD assay in rat plasma (*n* = 6).

Concentration (ng/mL)	Intra-day		Inter-day	
	Precision (%, RSD)	Accuracy (%, RE)	Precision (%, RSD)	Accuracy (%, RE)
0.64	7.10	4.30	4.80	3.30
1.80	6.20	-2.30	6.30	4.40
30.00	5.60	5.30	2.90	3.70
49.50	6.90	3.80	4.90	4.90

**Table 3:** Pharmacokinetic parameters of CAMD after intragastric administration of clopidogrel alone or coadministration of clopidogrel and XST to rats.

Parameter	Clopidogrel group(*n* = 6)	Combination group(*n* = 6)
T_max_ (h)	1.00 ± 0.85	0.75 ± 0.48
C_max_ (ng/mL)	8.92 ± 2.63	37.71 ± 6.34*∗*
AUC_(0-24)_	15.91 ± 2.93	84.13 ± 4.72*∗*
AUC_(0-*∞*)_	16.31 ± 3.15	107.03 ± 5.31*∗*
MRT_(0-24)_(h)	3.84 ± 0.94	6.39 ± 1.35*∗*
MRT_(0-*∞*)_(h)	4.43 ± 1.24	7.29 ± 1.40*∗*
Vd/F (L)	9.53 ± 1.21	11.26 ± 1.23
CL/F (L/h)	0.86 ± 0.21	2.04 ± 0.31
T_1/2_	1.79 ± 0.64	1.13 ± 0.47

T_max_(h): time to reach maximum concentration; C_max_(ng/mL): maximum plasma concentration; AUC: the area under the concentration time curve; _(0-*∞*)_: from time zero to infinity; _(0-24)_: from time zero to 24 h; MRT(h): mean residence time; Vd/F (L): apparent distribution volume; CL/F (L/h): apparent clearance; T_1/2_: the half-life of drug elimination during the terminal phase. *∗P*<0.05 compared with related parameter of CAMD in the alone group.

## Data Availability

The data used to support the findings of this study are available from the corresponding author upon request.

## Abbreviations

SD: Sprague Dawley
XST: Xuesaitong dispersible tablets
CES1A: Carboxylesterase 1 A
CAMD: Clopidogrel thiol metabolite derivative
UHPLC-MS/MS: Ultra-high-performance liquid chromatography-tandem mass spectrometry
MPB: The 2-bromo-3'-methoxyacetophenone.
